# The Need for Sustainable Teleconsultation Systems in the Aftermath of the First COVID-19 Wave

**DOI:** 10.2196/21211

**Published:** 2020-10-05

**Authors:** Guido Giunti, Richard Goossens, Antoinette De Bont, Jacob J Visser, Mark Mulder, Stephanie C E Schuit

**Affiliations:** 1 University of Oulu Oulu Finland; 2 TU Delft Delft Netherlands; 3 Erasmus University Rotterdam Rotterdam Netherlands; 4 Erasmus University Medical Center Rotterdam Netherlands

**Keywords:** telemedicine, COVID-19, telehealth, teleconsultation, exposure, software, digital health, organization

## Abstract

The physical and social distancing measures that have been adopted worldwide because of COVID-19 will probably remain in place for a long time, especially for senior adults, people with chronic conditions, and other at-risk populations. Teleconsultations can be useful in ensuring that patients continue to receive clinical care while reducing physical crowding and avoiding unnecessary exposure of health care staff. Implementation processes that typically take months of planning, budgeting, pilot testing, and education were compressed into days. However, in the urgency to deal with the present crisis, we may be forgetting that the introduction of digital health is not exclusively a technological issue, but part of a complex organizational change problem. This viewpoint offers insight regarding issues that rapidly adopted teleconsultation systems may face in a post–COVID-19 world.

## Introduction

The ongoing COVID-19 pandemic has dramatically changed the global landscape in general, and health care systems in particular. Over the past few months, varying states of lockdown have been declared worldwide [1], and teleconsultation has become the “new normal” way of accessing health care.

The social distancing measures that have been adopted worldwide because of COVID-19 [2] will probably remain in place for a long time, especially for senior adults, people with chronic conditions, and other at-risk populations. Teleconsultations can be useful to ensure that patients continue to receive clinical care while reducing physical crowding [3] and avoiding unnecessary exposure of health care staff [4].

Despite their promise, digital tools had proven difficult to implement until very recently [5]. Implementation processes that would typically take months of planning, budgeting, pilot testing, and education are now compressed into days. However, in the urgency to deal with the present crisis, we risk forgetting that the introduction of digital health is not exclusively a technological issue, but part of a complex organizational change problem [6].

In theory, all that is required for a telehealth visit is the right equipment and platform. In practice, the same barriers that existed in 2019 still exist today: proper protocols for patient care, equipment deployment, attitudes, and even legislation [7-10]. The lack of digital health training of health professionals is an increasingly recognized barrier. Although there are initiatives worldwide that provide specialization training on medical informatics (such as the American Medical Informatics Association in the United States, with nearly 1700 board-certified professionals [11], and England’s efforts with the Topol review [12]), Europe lags behind, with less than one-third of medical schools covering these topics [9].

## The Netherlands and Erasmus University Medical Center

On February 26, 2020, the first patient with COVID-19 was diagnosed in the Netherlands and admitted to hospital [13]. From then onwards, the number of infections, hospital admissions, and deaths due to COVID-19 infection increased rapidly, with a peak incidence of around 1500 daily new confirmed infections, over 500 daily hospital admissions, and close to 200 daily deaths two months after the admission of patient zero [14]. In that same two-month period, the Netherlands went into a state of “lockdown,” as did many other countries in the world. Standard hospital care was drastically reduced to deal with the surge of patients with COVID-19, especially in ICUs. At the time of writing, the Netherlands seems to have weathered this first wave of COVID-19, with current infection rates, hospital admissions, and deaths down to a trickle and lockdown restrictions increasingly eased.

Erasmus University Medical Center (Erasmus MC) played a central role in managing the COVID-19 crisis in the Netherlands by serving as the national coordination center for the distribution of patients with COVID-19 nationwide. The medical center is based in Rotterdam, and is one of the largest and most authoritative scientific university medical centers in Europe. Erasmus MC is a 1125-bed academic medical center offering specialized health care services in neurosurgery, cardiothoracic surgery, pediatric oncology, and neonatal and intensive care; in addition, it is a level I trauma center. A Microsoft-based, integrated electronic health record (EHR) solution called HiX (ChipSoft) has been fully deployed since June 2017.

**Figure 1:**
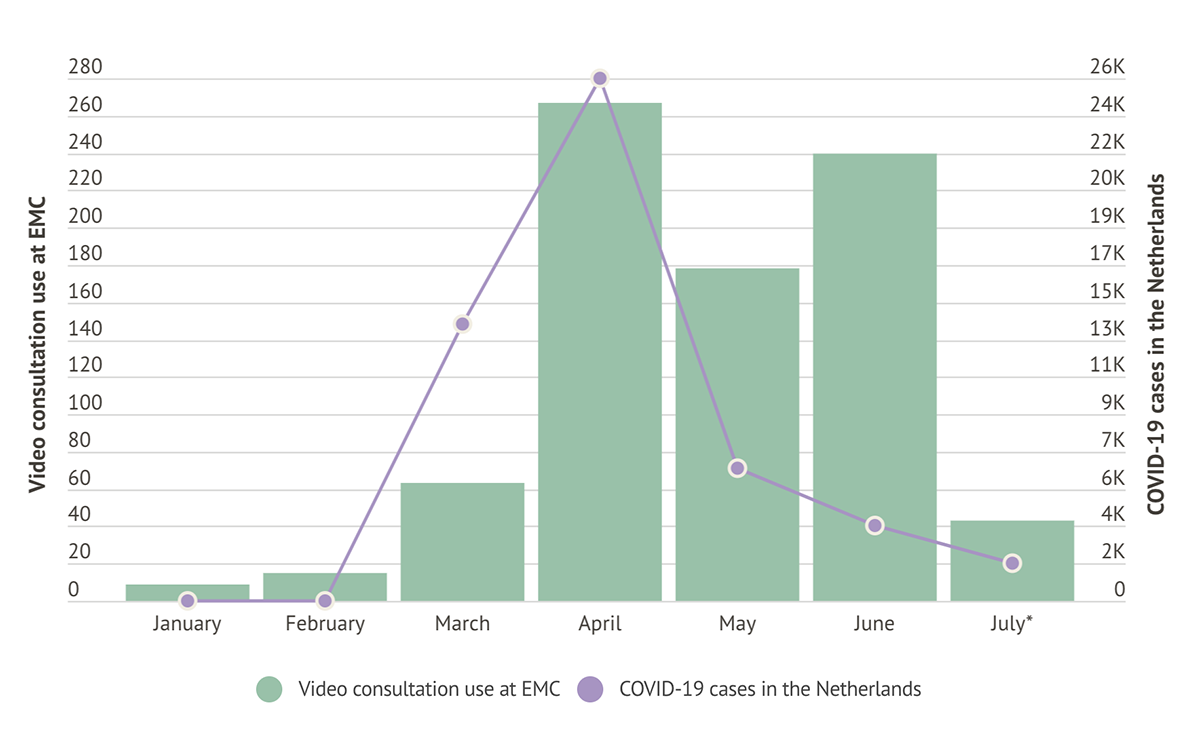
Video consultation use over time and COVID-19 cases in 2020. EMC: Erasmus University Medical Center.

As became common practice worldwide at the beginning of the COVID-19 pandemic, most of our clinical work had to be reorganized and priorities changed. In-person visits had to be postponed and nonemergency procedures rescheduled, with outpatient care shifting toward teleconsultation where possible. At that time, Erasmus MC’s teleconsultations occurred through telephone calls and an integrated EHR Skype for Business feature. Between March and June 2020, a clear spike in telephone consultations could be seen, from around 20,000 to an average of over 35,000. A similar fluctuation was observed for video consultations, which grew from approximately 10 per month to an average of over 187 (Figure 1).

**Figure 2:**
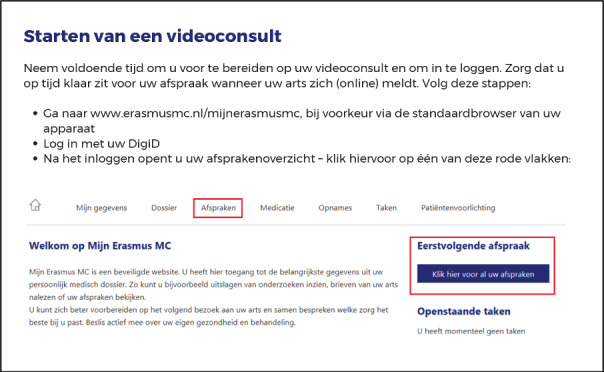
Didactic materials for patients.

**Figure 3:**
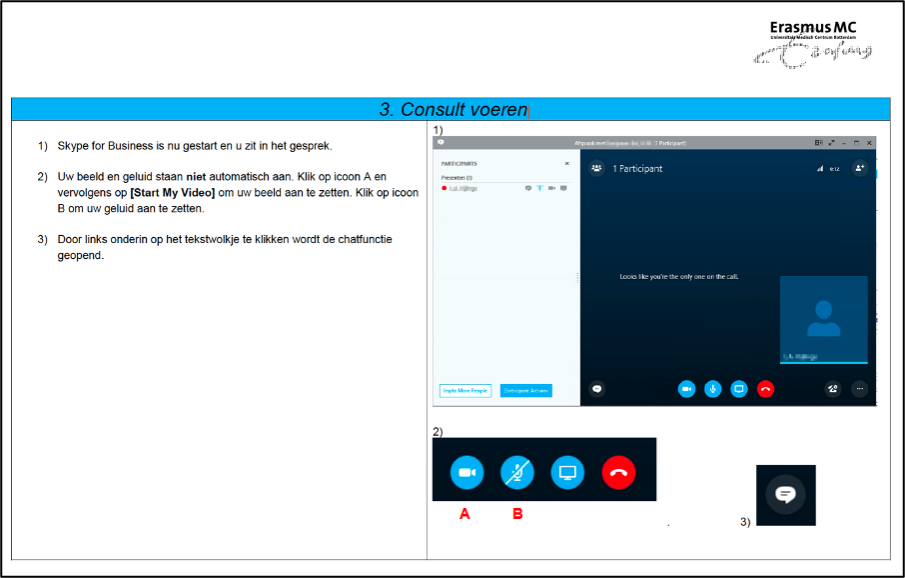
Didactic materials for health care professionals.

The rapid demand for teleconsultations required that more health care personnel be rerouted to cover the need, and steps had to be taken to increase the bandwidth of the service. It is in this context that our institution needed to develop an internal strategy for communication and training regarding the use of these digital tools that were not commonly used before the pandemic. To instruct staff and patients on the use of the platform, didactic material targeting the two groups separately was designed (Figures 2 and 3).

As seen in Figure 1, after the first wave of COVID-19, the number of telephone call consultations went back to pre-pandemic numbers, whereas video consultations have diminished but are still higher than before the crisis. Taking advantage of the benefit of hindsight, certain lessons have emerged from Erasmus MC’s approach to the sudden teleconsultation demand increase.

## Design and Implementation Considerations

Designing protocols for telehealth requires proper clinical and technical scoping with careful testing. Erasmus MC’s patients and health care professionals were able to adapt to the new teleconsultation approach, but attention should be paid to the care pathway flow. There are still concerns about how these tools could impact the professional–patient relationship [15], and the need to involve stakeholders in design and development is raised time and time again [16]. Even in situations where the urgency is great, implementation without the involvement of frontline care providers results in unanticipated incidents and disruptions to daily practice [17].

There are many other aspects that we must consider in this rapid implementation. Our choice of platform needs to be aligned with interoperability and security requirements such as EHR integration and General Data Protection Regulation (GDPR) or Health Insurance Portability and Accountability Act (HIPAA) compliance. Partnerships with new vendors should attempt to meet not only current but also future needs. Patient privacy and data ownership are still underexplored territories from policy and regulatory perspectives that need to be treaded carefully [18]. Patient-generated data is no longer just part of the health care system but rather belongs within a new context of “consumer” health care services [18]. Usage of data requires rights to be renegotiated where transparency and open dialog are paramount for a balanced agenda.

Returns on investment, consultation fees, costs, and telehealth rules in general are nascent at best, and in many countries, clinicians are not reimbursed for virtual consultations or online prescriptions [19]. Lack of reimbursement and revenue has been regularly cited as a significant barrier to the adoption and implementation of telemedicine services [20]. COVID-19 has been a great promotor for changes in this area. For example, in the United States, Medicare took an important step this past March to reduce the payment obstacle by covering telehealth in many more settings, at least temporarily [21].

Textbox 1 presents some important questions we need to be asking ourselves to prepare our teleconsultation system for a post–COVID-19 age.

**Questions for a post–COVID-19 teleconsultation system.**

- What are the advantages of developing a system in-house versus partnering with a vendor?
- How well can our institution’s current infrastructure handle in-house development?
- Will our institution benefit from an assortment of tools or a unified platform?
- What processes would need to be changed to accommodate a new teleconsultation system?
- How will the workforce be trained in the use of the teleconsultation system? What about the patients?
- What kind of reactions would adopting a teleconsultation platform generate?
- How will our institution communicate the policies and protocols for after COVID-19?

## Final Thoughts

It is imperative that the solutions we develop today attempt to be as sustainable as possible in the long run. It would be unfortunate if advances made today were to be discarded once the crisis is over. In this new era, the use of truly multidisciplinary teams that bring together not only health professionals but also designers, engineers, social scientists, and health economists, among others, is a critical first step in developing open, productive, and sustainable implementations [17].

We have the opportunity to start acting on a vision of the future of medicine, placing the building blocks for the consultation room of 2030. The impact the current pandemic will have on our society is greater than we can currently understand, and we must be responsible in our approach to the changes that are coming. Every step makes a footprint—let’s make ours count.

## Abbreviations

EHR: Electronic Health Records
Erasmus MC: Erasmus University Medical Center
GDPR: General Data Protection Regulation
HIPAA: Health Insurance Portability and Accountability Act
